# Supplementary material for: Evidence that injury can cause Drosophila gut differentiated, polyploid enterocytes to be recruited as stem cells via paligenosis
Source: bioRxiv. 2026 Feb 16:2026.02.12.705584. Preprint. [Version 1] doi: 10.64898/2026.02.12.705584 (PMC12934638; doi:10.64898/2026.02.12.705584)

bioRxiv preprint doi: https://doi.org/10.64898/2026.02.12.705584; this version posted February 16, 2026. The copyright holder for this preprint (which was not certified by peer review) is the author/funder, who has granted bioRxiv a license to display the preprint in perpetuity. It is made available under aCC-BY 4.0 International license.

Supplemental Figure 1.

A) Temporal expression of G-TRACE by switching Myo1A-GAL4/tub-GAL80ts *Drosophila* to 29° C for 7 days. Control (*top*) and Stress (*bottom*) with immunolabeling to show: GFP+/RFP+ cells (ECs) and GFP+/RFP− (cells derived from ECs) and lamin (purple) for nuclear membrane. Yellow carets (^) indicate diploid cells derived from ECs (i.e., GFP+/RFP− cells) after mitotic and oxidative stress. Such cells are not frequent in the unstressed control. Scale bar = 20 μm.

B) *Drosophila* midguts subjected to conditions depicted in panel A are plotted. Data points are individual organisms, ±SEM: two-tailed, unpaired Student's t-test. At least 4 independent experiments were performed. Genotype: w; Myo1A-GAL4; G-TRACE (UAS-nRFP, UAS-FLP,Ubi-63e>stop>nGFP)/tub-GAL80ts

C) Aged fruit flies (30 days old) have frequent diploid GFP+ (i.e., EC-derived) diploid cells relative to young adult flies (1-day old). Yellow carets (^) indicate diploid cells derived from ECs. Genotype: w; Myo1A-GAL4; G-TRACE.

Park et al., Supplementary Figure 1.

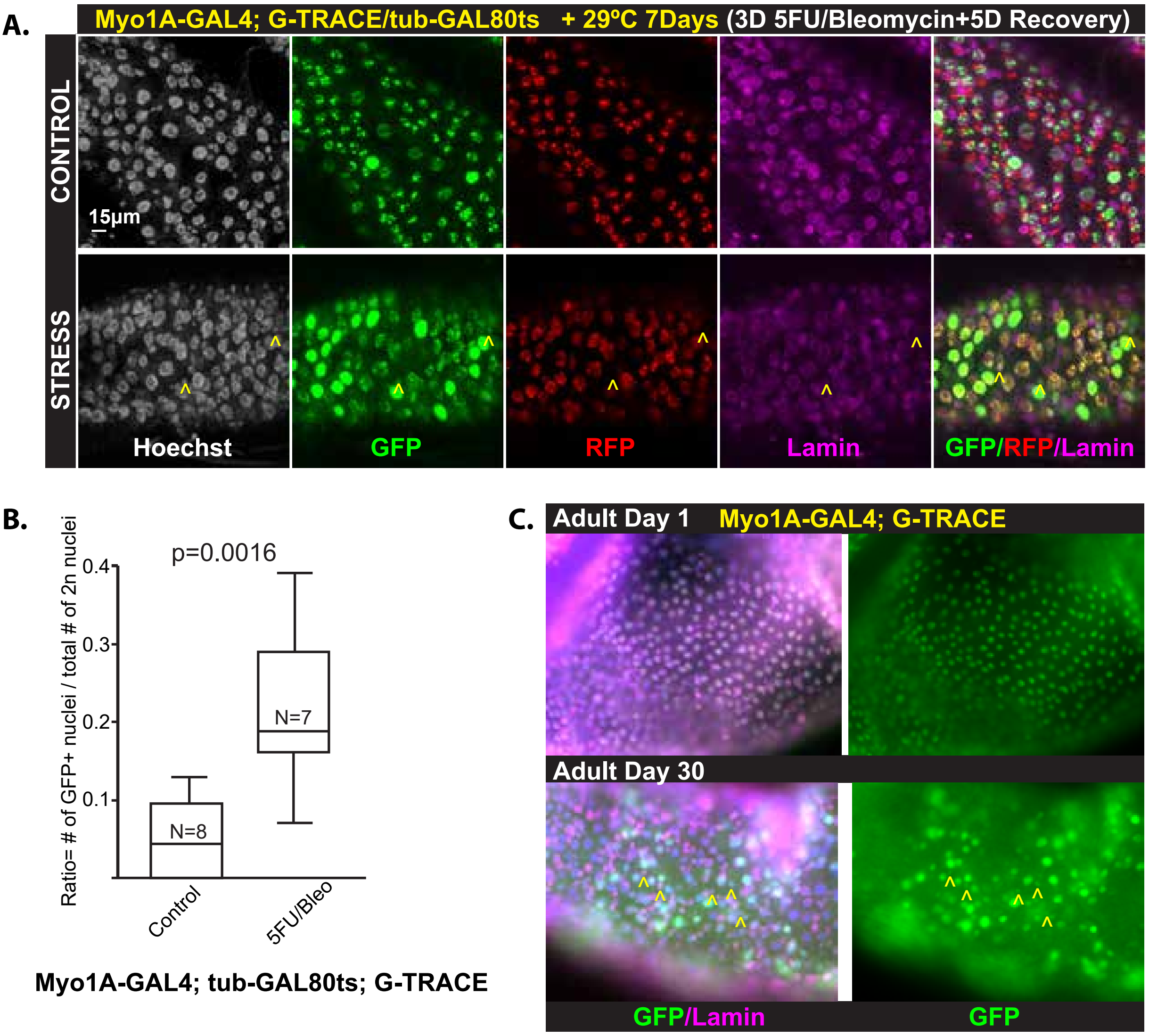

Supplement: Supplement 1 [file NIHPP2026.02.12.705584v1-supplement-1.pdf]
